# The house cannot be full: Risk, anxiety, and the politics of collective spectatorship in a pandemic

**DOI:** 10.1177/13678779211066330

**Published:** 2022-07

**Authors:** Tupur Chatterjee

**Affiliations:** University College Dublin, Ireland

**Keywords:** anxiety, bio-surveillance, contagion, film exhibition, multiplex, pandemic, risk, South Asia, spectatorship

## Abstract

This article charts the pandemic-engendered configurations of moviegoing cultures, leisure, and collective spectatorship in the Indian subcontinent and locates it within the discourses of personal risk, public anxiety, and industrial exclusion that have historically permeated the cinema hall. The pandemic marks a significant moment in the remaking of collective spectatorship and must be contextualized within the two-decades-long transition from single screens to multiplexes already under way in the Indian exhibition landscape. Through an account of the industrial developments in film exhibition in the last year and a half of pandemic time across two catastrophic waves of Covid-19, I offer some preliminary insights into the ways in which these shifts signal towards the cultural production of a new spectatorial body amenable to novel forms of bio-surveillance and datafication of self.

This article charts the pandemic-engendered configurations of moviegoing cultures, leisure, and collective spectatorship in the Indian subcontinent and locates them within the discourses of personal risk, public anxiety, and industrial exclusion that have historically permeated the cinema hall. The social act of moviegoing has always been an activity fraught with risk for several audiences based on their gender, sexuality, class, caste, and ability. Yet, the experience of being in the cinema theatre is also intensely tactile as it centres upon several excesses: swelling crowds, performative fandoms, long heaving queues, the *first-day first show* phenomenon, and the distinctive thrills of being engulfed in the chaotic frenzy. This is the familiar picture of the stand-alone single-screen cinema in India, which ruled its exhibition circuits till the advent of the multiplex. The multiplex arrived in 1997 and sought to define itself against the seemingly uncontrollable crowd politics of the single screen. It advocated for a stylized, privatized, cordoned off class- and caste-based experience of moviegoing, thereby marking the single screen as an inherently chaotic, unhygienic, and risky space which was *too public* – in other words, it was a space open to the working-class mass spectator, imagined to be the bearer of literal and figurative contamination. This article argues that the pandemic marks a significant moment in the remaking of collective spectatorship in the subcontinent and must be contextualized within the two-decades-long transition from single screens to multiplexes that is already under way in the exhibition landscape. ‘Risk’ – a category assigned to certain undesirable bodies – can no longer be controlled through a politics of exclusion, and now supersedes neoliberal cultural categories of class, caste, and gender.

The pandemic collides not only with the entirety of the ‘clean’, sanitized, and globalized multiplex assemblage, it also arrived at a time when India's screen and exhibition industries were already negotiating encounters with the digital via OTT platforms. The Indian scenario, then, is a part of the global retreat from theatres, along with an accelerated turn to digital platforms, which has led to a sharpening of the figure of a digitized spectator. Through an account of the industrial developments in film exhibition in the last year and a half of pandemic time across two catastrophic waves of Covid-19 in the subcontinent, I offer some preliminary insights into the ways in which these shifts signal towards the cultural production of a new spectatorial body, amenable to novel forms of bio-surveillance and datafication of self. Analysing press discourses, interviews, and publicity campaigns, this article maps how the major players in India's entertainment industries are crafting novel imaginations of a ‘safe’ spectatorial experience at the theatre. A delineation of these efforts, and the index of anxieties that they evoke, provides an entry into how the pandemic has altered the politics of public leisure, reconfigured the affective field of bodies and risk, and thereby remade the spectatorial imaginations of a global media industry.

While the pandemic is global, it must be contextualized within the local configurations of contagion and risk at specific sites. The site of cinema exhibition often mimics the symbolic qualities and anxieties associated with other spaces and boundaries – like the home – in the social life of a nation. Larkin (1998: 49) reminds us that cinemas are parochial, and must be studied through the emotional responses they create in a ‘sensory environment regulated by specific relations of lighting, vision, movement, and sociality’. While exhibition technologies may be transcultural, their impact on a specific location must be approached through an understanding of how theatres evolved within a nexus of prevalent social relations. The production of specific cinematic environments depends on ‘a negotiation between built space, the apparatus itself, and local social relations’ (Larkin, 1998: 48). Following these coordinates, this article expands our understanding of pandemic-engendered cinematic environments, and of the kind of idealized spectator at the heart of a new industrial diagram for contagion control.

In what follows, I sketch a brief history of fears around contamination and disease that have plagued cinemagoing as an act of leisure since its inception in the subcontinent. Next, the article looks at the arrival of the globalized multiplex in the late 1990s, after more than a decade of middle-class retreat from the cinema hall and into the world of television at home. Here, I also chart the key elements of the spatial politics of the multiplex marketed as a ‘safe’ class-based experience distinctly different from the ‘unhygienic’ single-screen cinema. The final section studies the collision between contagion and the material environment of the cinema theatre, and how it is leading to the rapid production of a predominantly ‘touchless’ encounter, available to a specific kind of digitized body. The article concludes with some preliminary thoughts on the future of post-pandemic cinemagoing cultures in India where new measures for surveillance and bio-policing redefine the conceptual boundaries of ‘safe’ pleasures and desires of public consumption.

## Contamination at the cinema: a brief history

One of the founding myths of cinema in the subcontinent centres on the apparent democratic space of the cinema theatre which houses a ‘national’ and united spectatorial collective. Yet there are few practices of collective leisure in Indian public life that have evoked a range of anxieties comparable to those associated with cinemagoing. The cinema hall can be read as a potent site for potential political and socio-cultural upheavals primarily because it inverts upper-class and caste-based ideas of public and private space. Rampant fears of miscegenation and contamination – both literal and figurative – have always been deeply embedded in the geography of the theatre. For over a century, for several kinds of spectators – based on their gender, sexuality, class, caste, and ability – the social act of moviegoing has always been a simultaneous act of embodying risk.

Historicizing fear and contamination at the cinema must be contextualized within the dominant structures of spatial organization prevalent at a local site. Indian middle-class notions of space and space-sharing have always stemmed from the conceptual boundaries between the ‘inside’ and ‘outside’ or the ‘private’ and the ‘public’. Gender and caste remain the two bedrocks of spatial segregation and the key sites that need seclusion against all kinds of contamination.

Several historians (Chakrabarty, 1991; Chatterjee, 1993; Kaviraj, 1997) have discussed how Indian elites appropriated and translated European concepts of private and public space. For instance, Kaviraj (1997: 84) reminds us that colonial modernity was premised upon a duality between the city and the country, where the former ‘was seen as orderly, hygienic, scientific, technologically superior and civilized’. Notably, an important distinction between European and Indian ideas of public space was that the concept of universal access did not exist among the latter. There was no place in the Indian cultural context that absolutely anybody could access at their pleasure. Colonial rule changed Indian elite encounters with the ‘outside’ – which remained threatening and unpredictable – but became a little less obscure. While the colonial state had little interest in creating inclusive enclaves for everyone it governed, the cinema hall quickly became a vessel for shaping spatial anxieties and encounters with the unknown-at-large. By the 1920s cinemagoing had become a wildly popular form of entertainment. Space-sharing at the cinema became unavoidable, and European and Indian ideas of public and private began to converge at the theatre.^1^

The risk of spreading disease and contagion, along with moral/sexual depravity, was traditionally associated with working-class men and *bazaar* publics.^2^ To this day, ideas around spectatorial taste, behaviour, and conduct at the cinema continue to be shaped by colonial discourses that originated in the 1920s. As Dass (2009) contends, elite Indians with access to education aligned themselves with refined tastes and ways of conduct, comparable to those of the British. The divisions were not so much between the colonizer and the native as absolute categories but between the upper-class and upper-caste educated Indians and the ‘mass’ of the illiterate poor. Using the findings of the *Indian Cinematograph Committee Report* of 1927–28,^3^ Dass argues that spectatorship, from its inception in India, was a site ‘not just of imagining community but also of asserting class difference and social hierarchies’ (2009: 79). Anxieties around health, hygiene, and safety are featured prominently in this seminal report. For instance, the British Social Hygiene Council visited India in 1926–7 and claimed that cinema was a major contributor in lowering the standards of sexual conduct and leading to an increase in diseases (ICC Report 1927–1928: 188, cited in Dass 2009)). Fears of miscegenation existed at two levels: between the colonizer and the native, and between the upper-class Indian and the subaltern mass.

The owners of India's first indigenous theatres belonged to the upper-class elite and were distinctly uncomfortable about opening their theatres to working-class Indians, whose bodies were imagined as repositories of ‘otherness’ and, by extension, disease and infection. For instance, Rustomji Dorabji, proprietor of several upmarket theatres in Bombay vehemently opposed any government decree that would require him to screen Indian films at his high-end theatres catering to the European elite and Westernized Indian middle classes. According to him:

If a theatre is asked to show even once a week one Indian picture, even that will ruin that particular theatre altogether, because Indian habits and the educated man's habits are so wide apart that with the betel leaves and other things which make them equally dirty and stinking, it will take another 3 weeks by the time you have cleaned it well and put it in order for the better class Indians.…

Once a theatre is spoiled, let me give you an example: I did show an Indian picture at my Wellington Theatre, *Lanka Dahan* … and I made Rs.18,000 in one week. But it ruined my theatre altogether.

I had to *disinfect* the hall and at the same time I had to convince my regular audience that I had disinfected it and so and so. Till that time, I went on losing money. (ICC Report 1927–28, 1928: 362–4 cited in Dass, 2009: 79, emphasis added)

Dorabji's comments remain eminently insightful in understanding the relationships between class, hygiene, taste, and public leisure in the subcontinent. Native audiences with a ‘taste’ for Indian films – simplistic, escapist, stunt-heavy, thrilling, and much inferior to Western counterparts – were also associated with a lack of social and physical hygiene, and their very presence as a collective sparked fear of an infected space. Writing on the history of cinemas in the Malabar region, for instance, Menon (2021) discusses how theatres were fundamentally miasmic spaces – a hotbed for the spread of epidemics and multiple contagions, including social and moral ones. She writes: ‘accounts of “miasmic” cinema halls construct the cinema hall as an assemblage of subaltern bodies and evince a fear of the “mass” as violent, amoral, contagious, and sensate’. Class, therefore, trumped race in the country's earliest experiments with mixed spaces for public leisure.

Srinivas (2000) notes that while the ‘public’ at the cinema hall was internally differentiated, a distinction existed between those who could enter the public domain as citizens of a political society and those who were the non-elite publics or ‘non-citizens’. Significantly, despite middle-class discomfort, the right of ‘non-citizens’ to access the theatre was never questioned. At the cinema – a peculiar harbinger of modernity entwined with technology – in theory, class, gender, and caste barriers were not applicable. The theatre management, however, adopted several methods to enforce different kinds of segregation that were ultimately closer to the cultural realities of spatial inhabitation entrenched in their local contexts.

The primary unease that upper-class Indian audiences expressed in their navigation of the single-screen cinema had to do with their classist and caste-based notions of security (mostly sexual security of Hindu women) and hygiene (centred on the eating and polluting habits of lower castes). Because the families that owned and built the country's theatres often belonged to the same class and caste status as the spectators, they were able to construct a space designed for several kinds of precautionary segregations (seating, ticket counters, ticket pricing, quality of chairs, bathrooms, etc.) to appease this middle-class uneasiness to an extent. What they could not control was who came to the theatre. An exclusion of working- or lower-class audiences would have been economically disastrous for both single screens and the film industry. Instead, they tried hard to ensure that within the theatre – despite inhabiting the same material environment – working-class men and upper-caste women shared as little space as possible.

Cinemas were architecturally designed with class and gender segregations in place. The cheapest tickets were for the stalls or the lower level, which had two sections, front, and back. The front stalls were notorious for hosting the most boisterous male spectators. The upper level was further subdivided into two: the Dress Circle, more expensive than the stalls, and the highest priced Balcony. Middle- and upper-class families, women, and children occupied the upper levels, while working-class men and a smattering of women filled the stalls. This was the familiar picture of the single-screen cinema hall for several decades post-independence. Most theatres also depended on their reputation for business. The class and caste status of spectators who frequented the theatre, along with its geographical location, infrastructural capacities, material comforts, and the kind of film it screened (Indian or/and Western) – determined its ‘reputation’.

While the cinema and its technological marvels were symbols of colonial modernity, from the 1950s to the 1970s, India's Art Deco and Modernist theatres were emblematic of a newly independent nation's architectural and infrastructural aspirations for public modernity. Cinemas were sites that gave form and shape to the citizen-spectator as a collective. Thus, despite risks and fears, moviegoing as the predominant form of public leisure was always also a site of intense pleasure, fandom, nostalgia, and freedom for many. It remains a meticulously tactile exercise as one interacts with the space through various kinds of touch: jostling against strangers, handling tickets that move from one set of hands to another, seats shared by many, eating, drinking, spillage, and public bathrooms, to list a few.

The Indian multiplex came in the late 1990s as an antidote to not only long-standing elite anxieties around miscegenation and contamination, but also tapped into middle-class desires for gentrification and the aspirational inhabitation of safe spaces. Notably, most multiplexes in India are located inside malls. In the last two decades, the multiplex has almost entirely eclipsed the single-screen cinema and come to determine the primary coordinates of film exhibition: economic, political, infrastructural, and socio-cultural. The mall-multiplex, as an assemblage, not only regulates class- and caste-based anxieties about sharing public space but also determines the entirety of the haptic field within which the spectatorial body must find its appropriate bearings.

The pandemic has vastly expanded the categories of risk: from marked and undesirable bodies to material surfaces and the very air we breathe in a privatized public space (ironically, the enhanced qualities of surface and air are the multiplex's primary attraction). The promised safety of the elite multiplex interior is now thoroughly compromised. Within this context, in the next section, I offer a short sketch of the multiplex as a built environment predicated on reassurance, and then delineate some of the preliminary contours of the present collision between contagion and surface.

## The Indian multiplex and the cultural production of safety

PVR Cinemas – owned by brothers Ajay and Sanjeev Bijli – is the country's first, largest, and most successful multiplex chain. The Bijli brothers, who were only tangentially involved in the exhibition business during the decades before liberalization, became the entrepreneurs responsible for India's first multiplexes in the South Delhi neighbourhoods of Saket (PVR Saket) and Vasant Kunj (PVR Priya) in 1997. In the last two and half decades, the multiplex has focused on not only changing cultures and habits of spectatorship but also producing new ways of being at the cinema. In other words, the multiplex has rewritten and recast several decades of spoken and unspoken codes of cinemagoing in the subcontinent by identifying persistent middle-class anxieties around gender- and class-based hygiene as the key site upon which to build new exhibition cultures.

India's first multiplex arrived in the middle-class neighbourhood of Saket in New Delhi, six years after the liberalization of its economy in 1991. It opened in a community centre – the name given to a cluster of small shops (sometimes around a cinema) – in one of the city's several centrally planned neighbourhoods. This community centre had a sordid cinema theatre called Anupam. By the mid-1980s, middle-class audiences had retreated from the cinema and were immersed in the pleasures of home video and satellite television. Jai Arjun Singh, a journalist and film commentator, who has lived in Saket since 1987, recounted in his blog, the momentous changes in the life of neighbourhood after the arrival of the country's first multiplex:

In the mid-1990s, strange things began to happen in our colony. Rumors grew of a light from the east, of a man named Bijli who had tied up with an Australian company to set up India's first ‘multiplex’ here. Rich relatives in other countries sent secret missives disclosing that multiplexes were cinema halls with *three or four screens* instead of one. We gaped in disbelief. Anupam shut down, then several months later we saw scaffolds and workers and large tarpaulins obscuring the building. In mid-1997 PVR Anupam opened, and I went to see the first film shown there, *Jerry Maguire*, nothing of which registered because I was too busy alternately leaning back in the plush sofa-chairs and sinking my feet into the carpeted softness of the floor. Things would never be the same again in our modest little Saket, which had, only 30 or so years earlier, been a forestland where men would go rabbit hunting. (Singh, 2005)

The multiplex thus became one among several nascent forms of globalized spaces that began to dot India's cities and towns. After the country's first multiplex had awed Delhi's middle classes, the Saket community centre came to be known as the famed PVR Complex. It began to change dramatically and rapidly. Fast-food chains like Pizza Hut, Barista, Café Coffee Day, and Subway knocked out the five or six scattered shops that had once populated it. Singh (2005) notes that in 15 years, the Saket community centre transformed ‘bit by bit, layer by layer, from a modest, bare-boned little colony center into a bustling hub of Delhi yuppie-dom’. The Saket success story was to be emulated all over the country. Coinciding with the beginning of India's post-liberalization consumer culture, the mall replaced the community centre, and several multiplexes-in-malls quickly began to populate India's urban horizons. They have since not only transformed landscapes but also entirely upturned the older temporal and spatial order of the cinema hall. The ATP (average ticket price) at a multiplex is 200 INR (it can go up to 1000 INR depending on the location and scale of multiplex), which is at minimum a 300–500% increase from ticket prices at the single-screen cinema.

Within a decade, PVR had managed to become the first film exhibitor in the country to secure private equity investment, expand to peri-urban and B-towns with the cheaper PVR Talkies, convert several old ‘heritage’ single screens to multiplexes, and expand to about 100 screens (PVR Cinemas, 2015). The following decade (2008–18) saw an exponential rise in their fortunes, focusing on cinemagoing as a technologically advanced luxury experience: with ‘7-Star’ theatres like PVR Director's Cut, and various kinds of digital cinemas like IMAX (Image Maximum) and ECX (Enhanced Cinema Experience). By 2018 the company was building theatres equipped with 4DX screens (which allows films to be augmented with effects like motion, wind, and scents) and moving toward the Superplex: 15-screen cinemas with virtual reality lounges, play areas and babysitters for children, gourmet dining, and a host of other attractions. In March 2020, PVR had 900 screens in 69 cities and was selling close to 1.45 million tickets annually (Bijli, 2021b). While these numbers are a drop in the ocean for a country as populous as India, what they indicate is that the multiplex ecosystem now owns the largest part of the existing pie.

The newly globalized middle class, with an ever-expanding disposable income, did not need much coaxing to shift their movie-watching habits from the 1000-seater single-screen cinema to the 200-seater multiplex. This was because they had already imagined this experience through what they were viewing on cable and satellite television in the 1990s. As in several other parts of the world (see Abu Lughod, 2005; Fehervary, 2013; Rofel, 2007), the Indian experience of globalization was felt strongly through the ubiquitous presence of television and advertising, which played a central role in the shaping of neoliberal desires and anxieties. As Mankekar (2012, 2015) has shown, after the privatization of the Indian economy, television shifted from capital goods to a heavy promotion of consumer goods, turning viewers and spectators into consumers. Television also played a key role in positioning India as an emerging market for commodities and accelerated its integration into global circuits of capital (Kumar, 2010; Mankekar, 2015; Mazzarella, 2003; Rajagopal, 1999). It was also clear that cable television programming and advertising had begun to slowly erase images of those who could not afford to consume, setting in process a politics of exclusion that was effectively carried forward by spaces like the mall and the multiplex.

Further, as scholars like Punathambekar and Kumar (2014: 2–3) have argued, television fundamentally re-mediated the private/public distinction. In the South Asian context, the material domain of the ‘public’ was one that was under colonial occupation, while the private sphere was always a site where concerns of community, nationalism, and solidarity played out. Here, the private and the public have always been intimately intertwined, and thus, as Mankekar (2015) posits, it was in the televisual home that people began to form new emotional convergences with commodities in globalized India. It was through the production of ‘commodity affect’ that television was able to create new regimes of sensoria: ‘domestic spaces became affectively charged spaces of consumption’ (Mankekar, 2015: 116). The desire for the commodity, however, went beyond just acquisition. Mankekar argues that desire in ‘commodity affect’ lies in the pleasures of gazing and longing for a commodity that one cannot possess but which helps them imagine the possibilities of life with it, or ‘what life could be like’ (2015: 115). These pleasures reflected the aspirations and fantasies of the viewers. The mall and the multiplex provided not only the material environments for these emotional convergences between people and consumer objects that began to take place with television at home, but also produced new temporal and affective entanglements with other infrastructures, and new refined sensory stimuli like sharp audio, air-conditioning (air), cushioned theatre seats, polished surfaces, and bright saturated colours.

Reflecting on the early years of the multiplex experiment in the country, Ajay Bijli, Chairman and Managing Director of PVR Cinemas, recounted in an interview (Bijli, 2021a):

I started the first multiplex in 1997 because from 1994–1997 there were no malls no shopping centers, so I had to pick up an existing cinema and carve it out into a fourplex. Delhi was my market. I was very fortunate that in every cinema I opened people liked good sound, good seating, hygienic atmosphere, and I didn’t realize that the appetite for people to watch movies was so massive. *So, all I had to do was create a very good conduit between the filmgoers and filmmakers, the infrastructure had to be made world-class*.

Other things I was noticing, McDonald's was there, KFC was there, *people were graduating towards a very clean environment … an ecosystem was forming around it … so my timing was okay*. (Emphasis added)

As Bijli's comments underline – the mall-multiplex – fused in an unbroken signage of new technology, new architectures, and new infrastructures was able to capitalize on several pre-existing middle-class and upper-caste neuroses and market them effectively packaged as national progress, innovation, and ‘world-class’ infrastructure. These are the expressions frequently used to describe the malls and multiplexes as hallmarks of metropolitan India's quick march toward a global city template. As I have also argued elsewhere (Chatterjee 2018), an interrogation of the industrial design of these places reveals that all of it is entirely predicated upon a visible reassurance of safety and hygiene, in a manner that can ensure the constant presence of the middle and upper classes. Further, Bijli's ‘timing was okay’ as the Indian audience had already been primed through a decade of immersive cable and satellite television. Television had remade the home as a key site for media consumption. The multiplex thus, first and foremost, sought to expand the affective qualities of the televisual home.

A multiplex development arc that is comparable to the Indian scenario is that of Singapore. Ravenscroft and Neo Wee (2001: 119) argue that the spatial metonymy of the cinema within the larger matrix of the mall is a way to reassure young people about consumerism as a central tenet of contemporary Singaporean society. A part of this is Singapore's larger residential redevelopment and the highly regulated nature of the urban environment in the country. The sanitized and planned environs of Singapore have long been objects of aspiration and envy for India. Therefore, in their design and core ethos, Indian multiplexes have heavily borrowed from Singapore. Govil (2015: 133–4) points out that the multiplex in India marks ‘both a portal into the west and a gateway into the globalization of Indian life’. He reminds us that consumption and commercialization are the twin logics of the multiplex, which seeks to create a cordoned off zone merging three spatial utopias: the urban exterior, the theatrical interior, and the on-screen space.

PVR Cinemas, for instance, uses the term *multiplex culture* to broadly define their integration of neoliberal film spectatorship with specific design elements. According to Ajay Bijli:

PVR has played a catalyst in bringing the ‘multiplex culture’ to India. We try to leave no stones unturned to provide the next level of cinematic experience to our patrons. One can easily experience it through the grand architecture, design, and facilities provided at our properties. (PVR Cinemas, 2015)

A key aspect of India's multiplex culture, thus, is the cultivation of spectatorial practices that consciously distance themselves from the stand-alone single screen and its experiences. Thus, at the surface level, multiplex culture prioritizes a celebration of visible hard infrastructures like new film projection technologies and enhanced audio, along with soft infrastructures like interior design and architecture. Everything is promoted and packaged as convenience: it is a place saturated with several bright and often interactive screens guiding spectators to manage their inhabitation of the space without needing much human assistance. Yet – in keeping with upper-class and caste expectations – it is also overwhelmingly concerned with service and hospitality.

The pandemic collides with the entirety of this assemblage, potentially ‘polluting’ an erstwhile impenetrable ‘hygienic’ fortress cemented by the politics of exclusivity. The current situation also intersects with the social divisions prevalent in the country. As Jaaware (2018 cited in Menon, 2021) has argued, structurally, caste and class in India are often more concerned with figural touching than literal touching. The pandemic overturns this dynamic as the site of fear has now decisively moved to literal touching. The second wave that swept through the country in April–May 2021 threw the middle and upper class off their perches of class- and caste-based insulation. They became disease-carrying bodies despite living and moving within closed, ‘clean’ enclaves. Such a cataclysmic event, then, not only necessitates a recalibration of existing structures but also provokes newer ways of understanding risk and risk mitigation. Next, I turn to how some cinematic environments are shaping themselves to align with this altered ecology of anxiety.

## Reconfiguring body and risk

As India slowly recovers from the tsunami of the second wave of Covid-19, and predictions of a third wave loom large, it remains too early to map the total coordinates of pandemic-induced changes in the country's moviegoing practices across habit, form, and infrastructure. However, there is an emerging picture of how India's multiplexes are negotiating the reconfigurations of body and risk in an era where excesses and panic cannot be kept at bay with class- and caste-based barriers to entry. The pandemic also reorders the relationships between local and nostalgic practices of cinemagoing and the global protocols of the ‘new normal . Ultimately, the pandemic has accelerated what was already in motion in India's neoliberal imagination of a new digital spectator and the kinds of ‘smart’ spaces they should access for leisure.

India's existing single screens, for instance, were reeling under the multiplex onslaught in the last decade with several going out of business after losing middle-class patronage. The decline of the single screen began in the 1980s with the arrival of television and home video leading to a middle-class retreat and the multiplex followed in the late 1990s. A recent report in NPR (July 2021) – shared nostalgically and widely on Instagram and Twitter by multiplex goers – discusses the ‘dying out’ of several single screens during the pandemic. According to this report, the number of single screens in the country fell from 10,000 to below 7000 between 2010 and 2019. The pandemic has wiped out scores of others in the past year. Unlike the multiplexes, which despite heavy losses were still able to stay afloat through bank loans and liquidities, single screens, trapped by heavy taxes and government regulations, were forced to shut up shop for good. Several single screen owners also cite the easy availability of video-on-phone and OTT platforms as huge barriers to attracting crowds to the cinema. The near erasure of the single screen from the country's exhibition terrain also means that working- and lower middle-class populations are further exiled from a predominant site of leisure and marginalized in the industrial imaginations of the country's current spectatorial collective.

The multiplex reads this moment very differently. PVR Cinemas suffered losses close to $ US80 million and laid off 9000 of their 15,000 employees. All cinemas closed for seven months, from March to October 2020, and again from April 2021 to the time of writing this article – July 2021 – with some states allowing theatres to open with 50% capacity. India's major film companies have held back most of their star-studded big releases, while some films with major male stars like Akshay Kumar (*Laxmii*; dir. Lawrence, 2020), Salman Khan (*Radhe*; dir. Deva, 2021), and Dhanush (*Jagame Thandhiram*; dir. Subbaraj, 2021) have seen a direct-to-streaming release – a historical first for a country driven by the ‘first-day first show’ phenomenon. Cinemas opened in October 2020 for a few short months. Multiplexes reopened with reduced ticket pricing, re-releasing older films, and widely advertising their advanced Covid-19 safety protocols. In March 2021, shortly before the second wave engulfed the country, younger and upcoming Bollywood stars like Janhvi Kapoor and Rajkumar Rao participated in a short-lived ‘return to theatres’ campaign to promote their horror-comedy *Roohi* (dir. Mehta, 2021), the first Bollywood film to release theatrically in a year. Several of their colleagues with upcoming releases photographed themselves at PVR's multiplexes and posted Instagram stories about the joys of returning to the theatre, emphasizing how safe they felt at the multiplex.

After the first wave, which was grim but not as catastrophic as the second, multiplex operators seemed upbeat. In several interviews between October 2020 and March 2021 with the English and Hindi language press, PVR Chief Ajay Bijli talked at length about why he remained hopeful about the future of multiplex moviegoing in India. In an interview with CNBC International TV, he said: ‘80% of our consumers are below the age of 39. They are resilient, and they cannot be incarcerated at home. The human body is not designed to remain under a lockdown at home’ (Bijli, 2021c). Given that Indians do not have too many other forms of ‘social outing’ like theme parks or baseball games, they spend their leisure time at the movies. In another interview with journalist Shoma Chaudhury (Bijli, 2021a), Bijli denied charges that multiplexes have made filmgoing an elite pastime and said that he has several multiplexes in small towns and cities where people can experience ‘a very clean, hygienic atmosphere for 100 rupees. We, exhibitors, were very keen that at every price point there should be a hygienic, high-quality cinema experience.’ However, as discussed above, conceptual boundaries of hygiene, cleanliness, and high quality remain deeply mired in class and caste biases in the subcontinent.

The multiplexes also command almost 60% of the country's box office revenues and thus are not overtly fazed by the rapid rise of India's digital platforms, even though the major prestigious players have an extraordinary number of paid subscribers, with Disney Hotstar at 300 million monthly subscribers, Amazon Prime Video at 13 million, and Netflix at 11 million subscribers. These numbers increased substantially during the pandemic. Along with building massive libraries of film and television content, these platforms are also investing in producing ‘original’ content at a rapid pace. Several prominent players from India's mainstream film industries – producers, actors, directors, technicians, writers – now simultaneously work for both digital platforms and for films meant for theatrical release. This has been aided by a significant rise in the number of smartphone users in the country – from 127 million in 2012 to 478 million in 2018, expected to reach 900 million by 2022 (Sun, 2021). Further, the rise of local language internet users in the Indian context – 42 million in 2011 to 234 million by 2016 – signals the arrival of vernacular practices that forcefully challenge Anglo-centric versions of digital cultures (Punathambekar and Mohan, 2019). The Indian state is now one of the strongest champions of digital and has actively supported several private players who are buying internet infrastructures.^4^

Bijli and his team see this as an aberration and not particularly different from the ‘biggest threat to cinemagoing’: television at home (Bijli, 2021b). Box office releases determine the monetization roadmap for films at large. Depending on their performance at the box office, they then move to digital platforms and television channels. They are bought and sold at prices determined through this initial box office levelling. Citing India's ‘insatiable appetite for the movies’, Bijli and his team began a recovery process for the multiplex chain through the ‘PVR Cares’ campaign: a visually stunning technical walkthrough of measures the company has taken to control contagion and contact at their theatres, to ‘win back the consumer's trust’ A quick analysis of the campaign illuminates the presence of a sharply digitized spectator-consumer body at the centre of it.

In a 4 minute promotional video (PVR Cinemas, 2020 ), we see visuals of PVR multiplexes polished and scrubbed like never before. An American accented male voiceover introduces us to the protocols in place: the box office cashiers are socially distant behind safety shields, ticketing is mobile and contactless, with PPE kits and single-use 3D glasses available at the ticketing counters. Physical and pat-down security searches are replaced by a digital thermometer screening and a status check on the government mandated *Aarogya Setu* application, which is built for ‘contact tracing, syndromic mapping, and self-assessment’ and can only be downloaded on smartphones. The staff has doubled down on cleaning using a variety of disinfectants that belong to the new dictum of contagion: anti-bacterial microfilms on surfaces, electrostatic sprays on all surfaces, air quality and humidity checks, sterilized food packaging, crockery, and cutlery. PVR staff and employees are subject to constant health screening and Covid tests after surveys revealed that spectators feared the spread of infection via workers. Touch, thus, now arrives via technology: ‘We have modified our procedures using technology for seamless, yet personalized service. With digital payments and non-invasive thermal screening, the touch of care replaces the physical one’ (‘PVR Cares’ campaign copy, PVR Cinemas, 2020). The ‘safe’ spectator must then not only be completely at home in ‘touchless’ environments but also somewhat of a techno-evangelist acquiescent to various forms of bio-surveillance and datafication of the self to participate in this new regime of what constitutes the ‘hygienic’.

These measures were poised to be successful before the second wave. After *Roohi* was released on 11 March 2021, two major delayed Bollywood mammoths *Sooryavanshi* (dir. Shetty, forthcoming) and *83* (dir. Khan, forthcoming) were slated for release in end-March and April 2021. The ferocity of the second wave derailed these plans, with these films being postponed indefinitely and India's multiple film industries having to halt all production once again. As noted, the second wave jostled upper-class Indians out of their privilege-based safety. In an interview with *Fortune India*), Ajay Bijli (2021b) discussed some potential long-term changes to the exhibition business. Noting that the second wave has affected middle-class morale, he said:

Confidence level of people has gone down dramatically, we were happy when we opened up, there was a false sense of security the entire country got, no reason to feel secure, vaccinations had not happened still we found that people came in hordes to cinemas.

Emphasizing vaccinations and more digitization, Bijli also revealed that the company was planning to remove physical box offices entirely, or convert them into food and beverage spots. There are also talks of building gaming facilities and spaces for live performances to optimize space at the theatres.


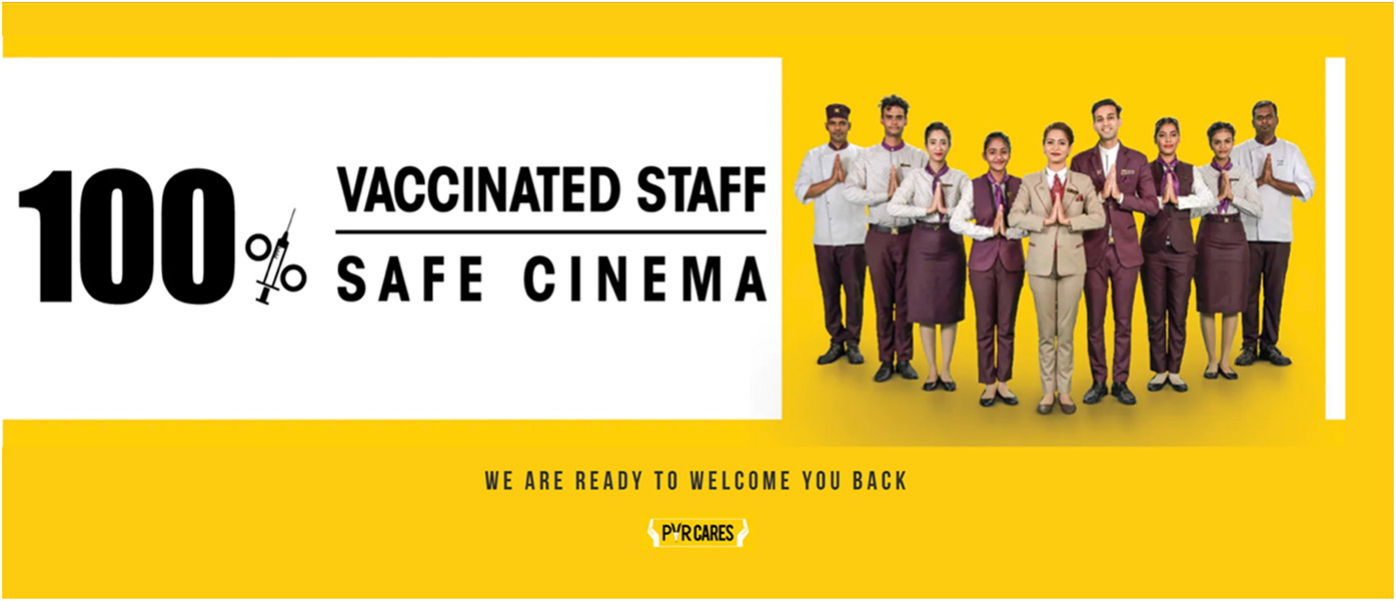


‘PVR Cares’ campaign website.


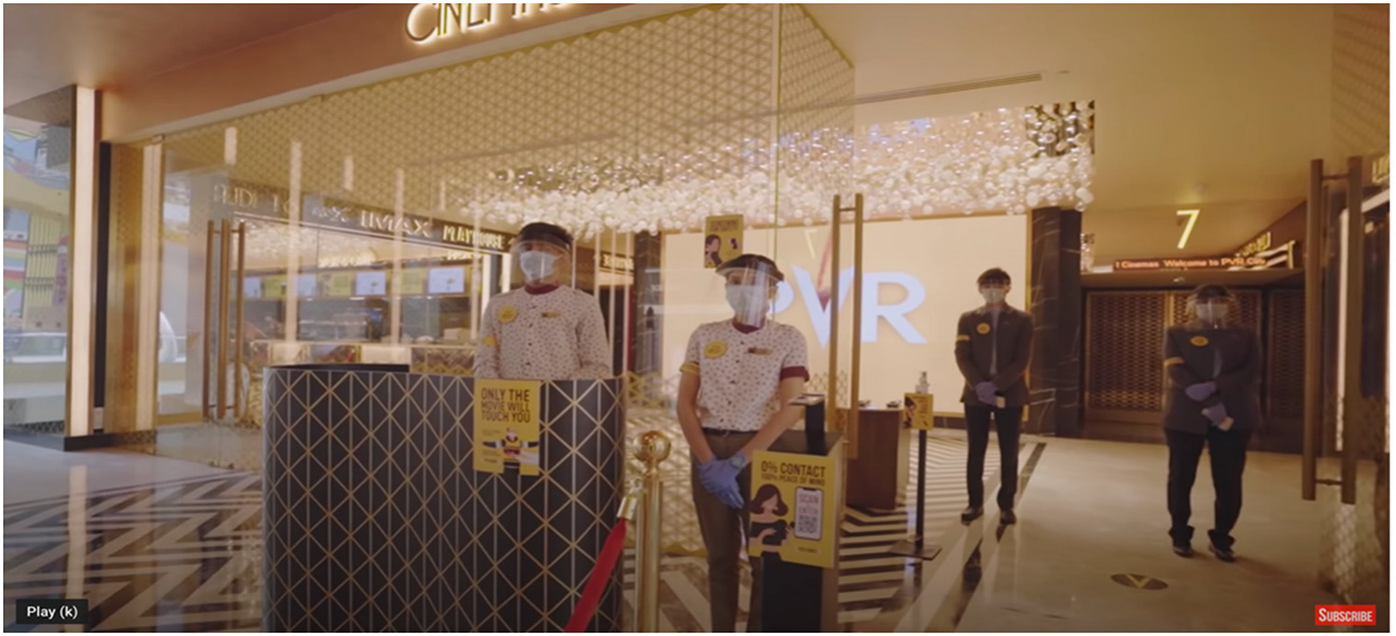


Screengrab from ‘PVR’ Cares promotional video.


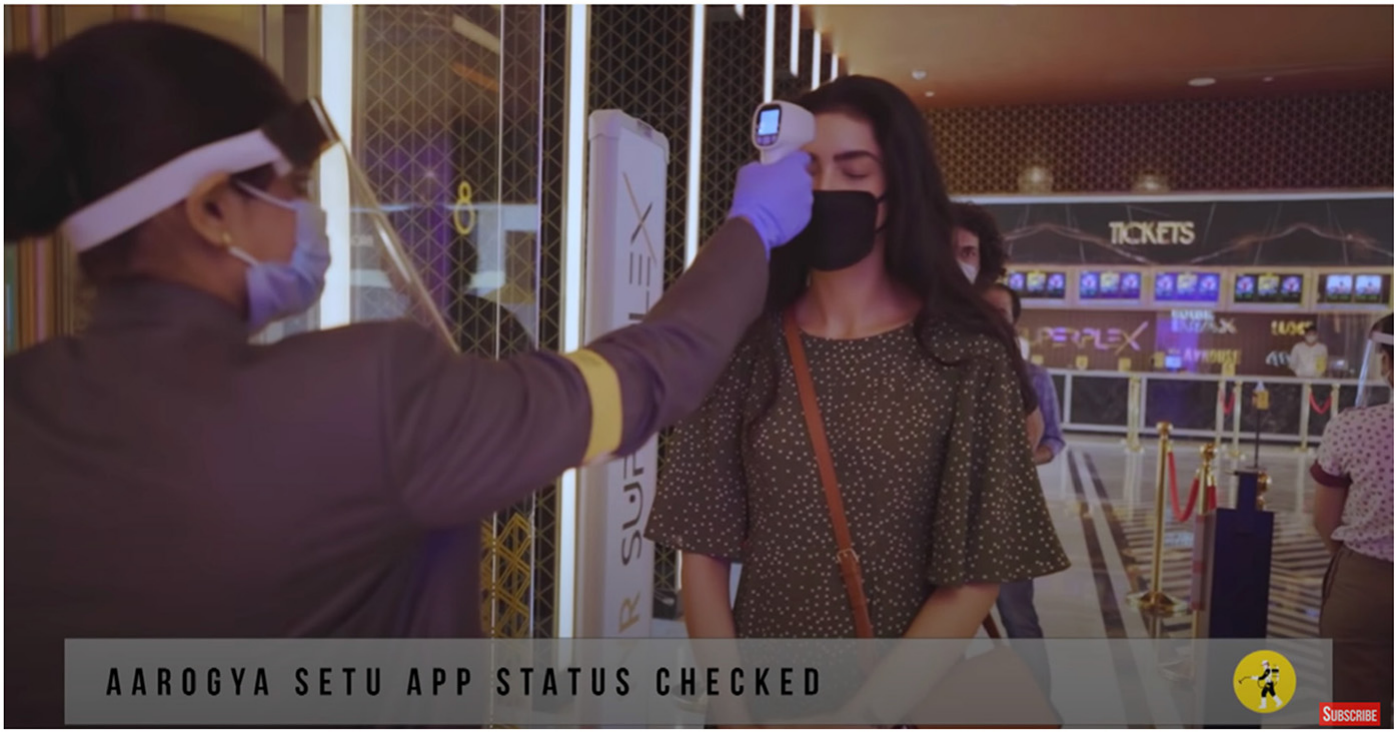


Screengrab from ‘PVR Cares’ promotional video.


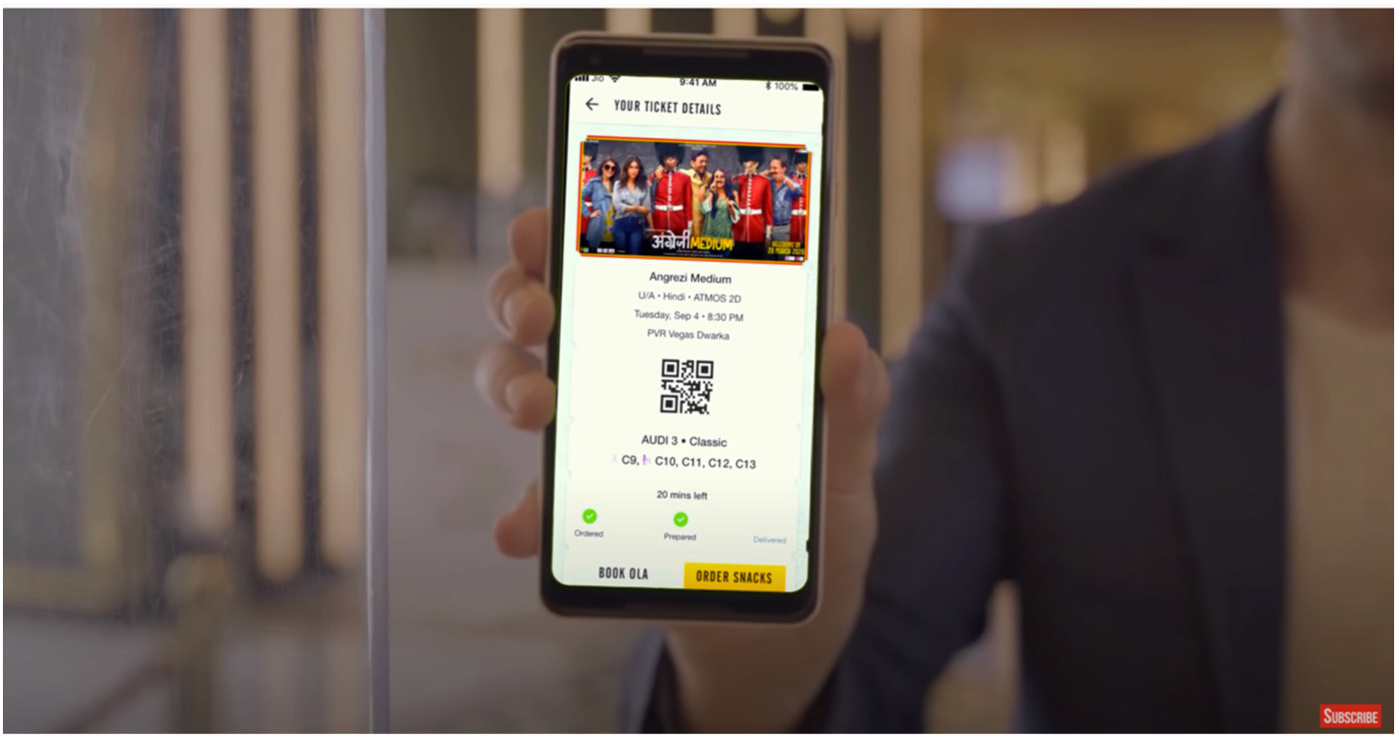


Screengrab from ‘PVR Cares’ promotional video.


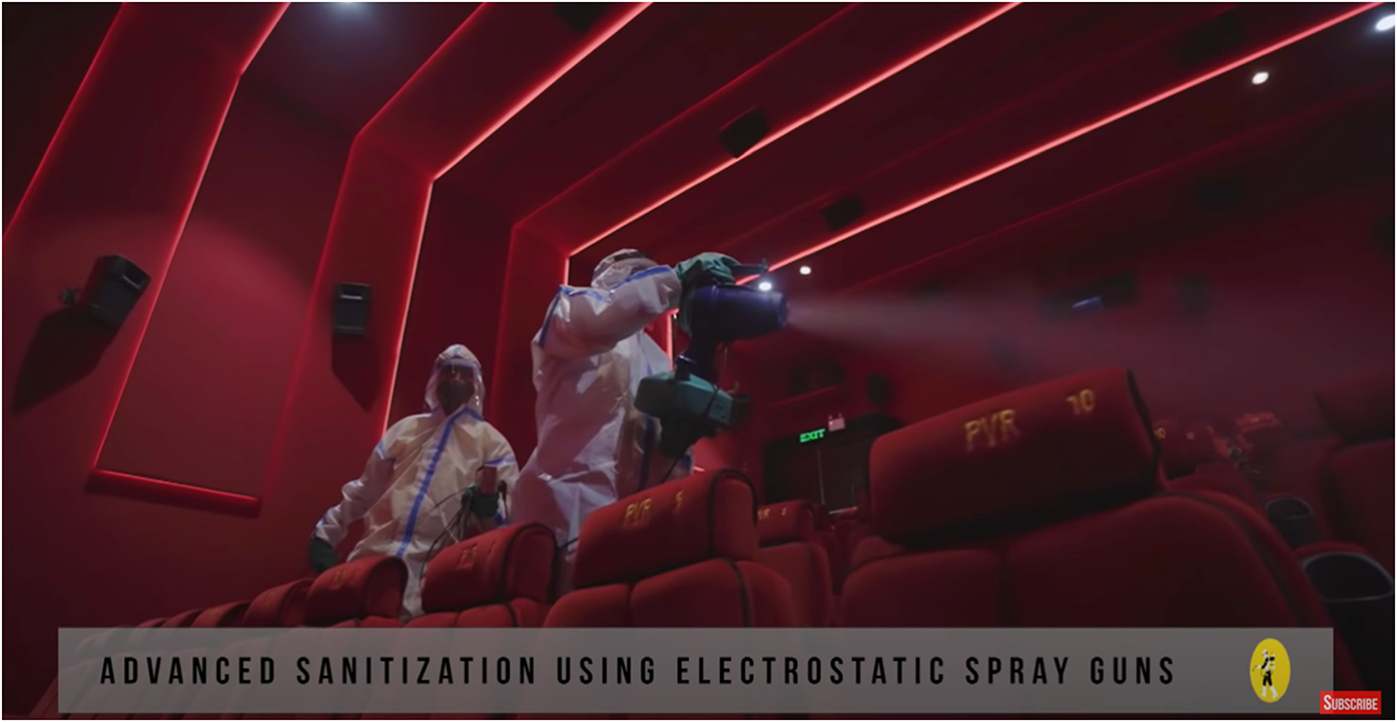


Screengrab from ‘PVRCares' promotional video.

In the pandemic-induced spectatorial regime, the hapticity of the digital viewer is redirected towards the cinematic spectacle. To compete with the enhanced potentials of home viewing, both film and exhibition industries are moving towards the ‘big event’ film. This usually signifies a big budget film with major stars released during a prominent festive holiday. For instance, *Sooryavanshi* (dir. Shetty, forthcoming) – a Bollywood *masala* starring three big male stars – was finally released on 5 November 2021, after being stalled for two years. It was the first major mainstream release after the second wave, poised to attract a big audience. The film was not released on a streaming platform. Shortly before the film's theatrical release, its male leads, Ajay Devgn, Akshay Kumar, and Ranveer Singh came together for a short promotional video. Standing together in a multiplex, they appealed to the Indian viewer's spatial nostalgia for the big screen: ‘Friends, do you remember this place?’ Calling the pandemic ‘an interval’ in the relationship between cinema and spectator-in-the-hall, the stars enticed Indian audiences to come to the (multiplex) theatre with their families for this Diwali special release. PVR Cinemas matched this fervour through new social media promotional campaigns underlining that going to the cinema was now as safe as going shopping and eating out, thus issuing a ‘public’ invitation to partake in the enhanced pleasures and desires of consumption, but within a new cultural regime of safety via bio-surveillance.


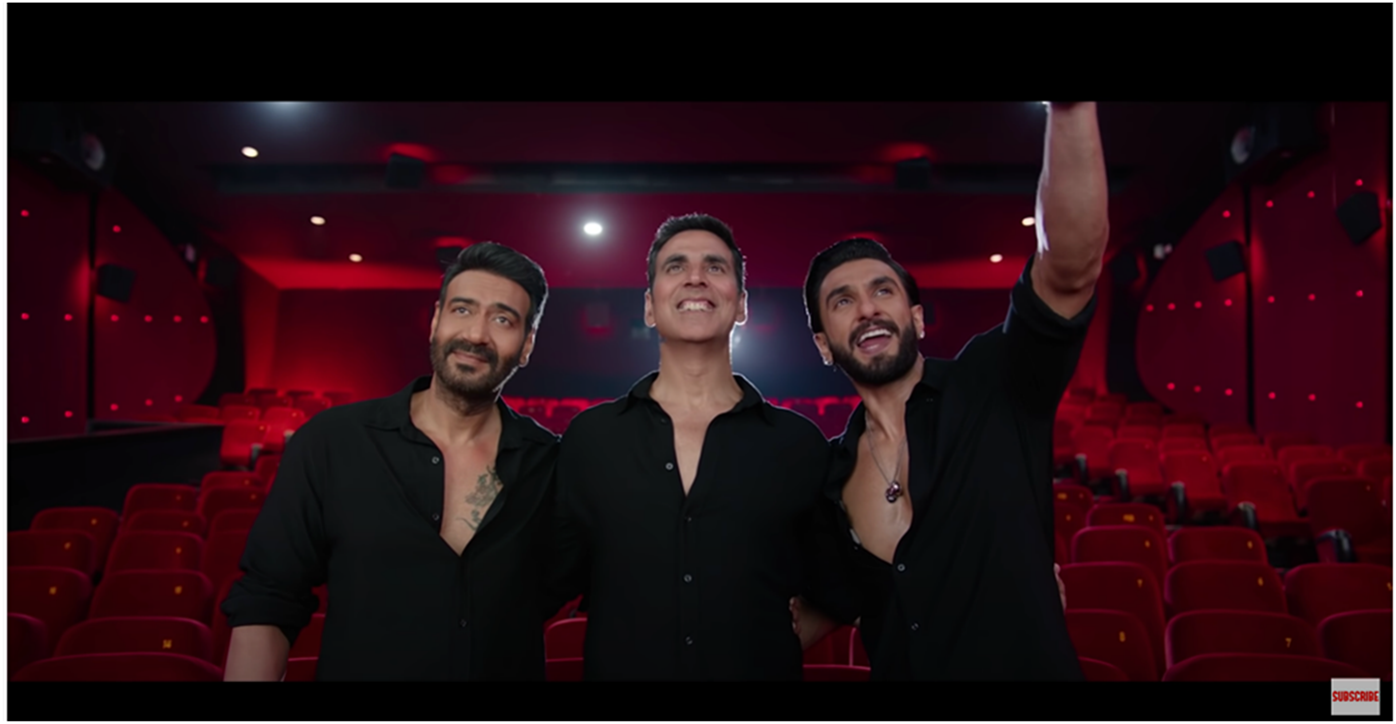


Ajay Devgn, Akshay Kumar, and Ranveer Singh promoting *Sooryavanshi* in a multiplex (screengrab from T-Series promotional video, 2021).


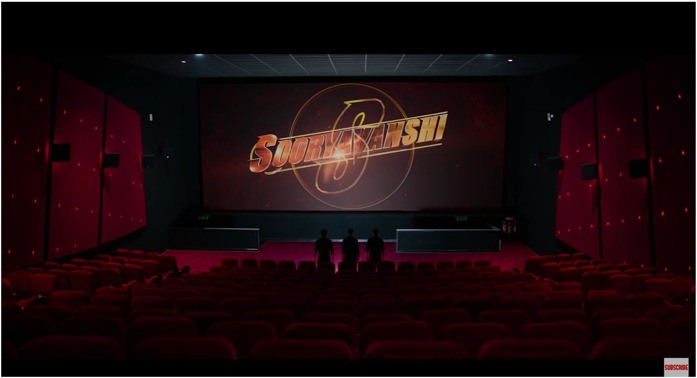


*Sooryavanshi* promotions: evoking the pleasures and desires of the cinematic screen (screengrab from T-Series promotional video, 2021).


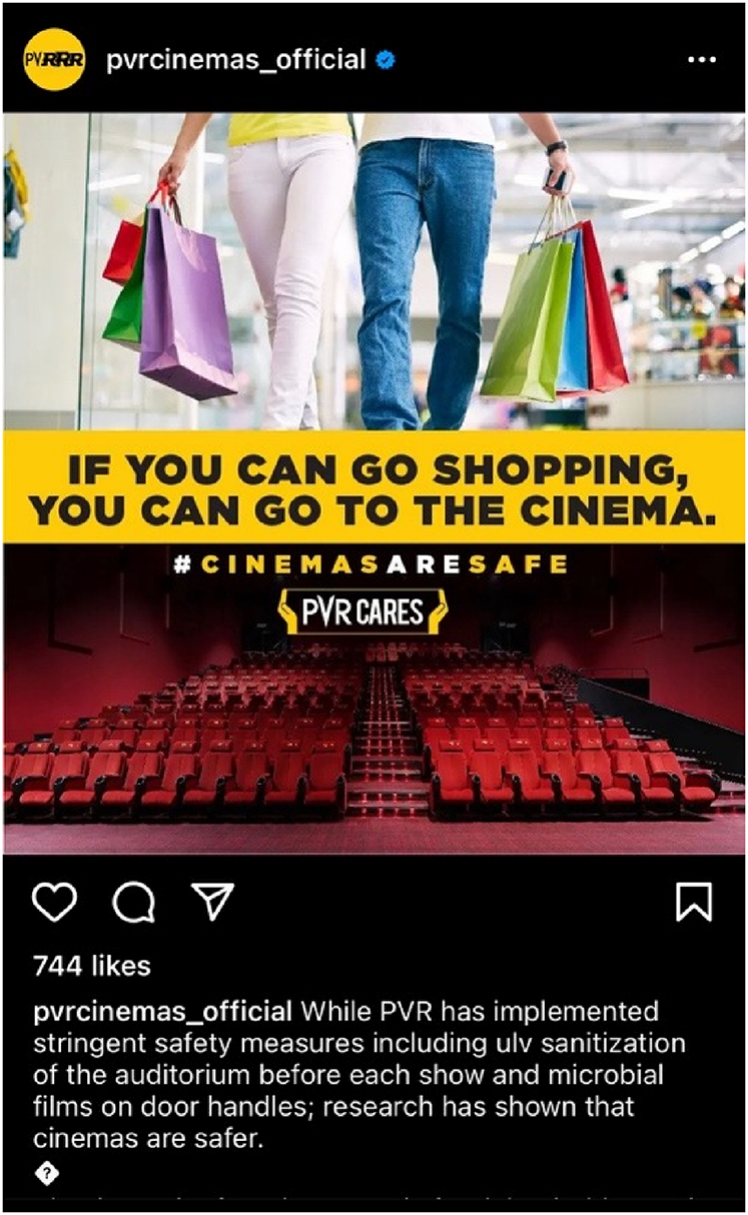


PVR Cinemas promotional material from their social media handles.

Moving away from solely a film exhibition model, the company now seeks to produce a new and enhanced out-of-home entertainment experience. As Bijli (2021b) put it:

We have to work very hard on becoming more and more experiential, it will have to be like Disney, what Cirque du Soleil did – it has to be very experiential when people go out not just from a technological point of view but also human interaction people must feel very very good when they go to the cinemas and think that this is something different: when I watch a movie at home it is utilitarian, but when I go out it's truly experiential.

Cirque du Soleil, cited as an example of a potentially new form of theatrical experience, merges Disney animation with live-action acrobatic performances. While this may seem radical in the Indian context, it is an advancement of the multiplex's initial ambitions to craft a total spatial experience as *the* event, one that exceeds the film text. Bijli's thoughts are echoed by the company's CEO Dutta (2020):

The fundamental question is what business are we in? And we’ve checked with consumers as well and while we do show movies and we are in the business of movies but the industry we are catering to – we’re in the business of out-of-home entertainment and really that pie is very big, and the question is who would have ever thought that F&B (food and beverage) sales for a cinema company would be 1000 odd crores? Who had thought that a media business would touch 400 crores of advertising on screen?

A decade after the multiplex had arrived, in a 2007 interview, Dutta (2007) had already hinted at the primary ethos of the multiplex as more than a new form of film exhibition in India:

Because the product that we peddle is not our product. It's someone else's. So, we are really like a box in which the cake goes. We are really like the packaging … in which the cake goes. We say that we are not in the exhibition industry we are in the experience industry, and experience really cannot be marketed. It needs to be felt. The whole idea is a delight factor. So, what we say is we are seduction marketers. We are here to seduce.

The company is on its way to building new pilot theatres, which will be completely redesigned touchless experiences and ‘as futuristic as we can make them’ (Bijli, 2021b). Such spaces inherently anticipate spectators on the right side of the digital divide, vaccinated and antibodied, completely at home with a variety of smartphone applications, and pliable to various forms of digital attendance and presence before, during, and after inhabiting the cinema. Contagion thus merges into renewed class-based fantasies of safety through hyper-technologized surfaces that can offer reassurance while still centring the pleasures of public consumption.

## Conclusion

Control at the cinema hall is not novel. Since their arrival as sites of collective leisure, at every moment in history, there has been a tension between industrial design and spectatorial behaviours. The single screen remains metonymous with a space where behavioural excess transcends architecture and design. The multiplex sought to invert this : spectators must be ordered via design. Single-screen cinematic cultures were unpredictable because they were subject to variants like the ‘reputation’ of the hall, weather, festivals and holidays, projection and sound technologies which differed greatly across theatres. They were also located on the street, at the centre of urban topography. Crowd formations, their performative fandoms, and control measures deployed by the authorities depended on these variables. The multiplex – at its core – seeks to erase all these discrepancies. Certainties across design, sound, projection, seating, temperature, colour, food, branding, and a heightened class awareness are meant to contain the spectator-consumer from spilling out of a specific pattern.

The pandemic greatly heightens these impulses as the ‘risk’ is now invisible and potentially everywhere. While it does act as a kind of ‘leveller’ – as the disease-carrying body can now be anyone – it also strengths the politics of industrial exclusion. Neoliberal spectators are expected to acquiesce to this new spectatorial regime where their data is sought for their own good. They can once again partake of the pleasures of ‘public’ consumption in the company of other digitized spectators who are proficient in accessing a touchless, ‘smart’ space. The corporate work of contagion control thus becomes an expanded avenue for newer forms of border-policing sites for collective leisure.

## Notes

1. See Arora (1995) for an example of how colonial anxieties around the native gaze found expression in regulating the sartorial choices of white women at the cinema.

2. Chakrabarty (1991) for instance, discusses the *bazaar* (marketplace), a literal and imaginative site that firmly belonged to the public and the outside – an ideal space for transgressions, border crossing, dirt, disorder, and contamination.

3. The Indian Cinematograph Committee was established by the colonial British government in 1927 to study the social, political, and cultural effects of cinema in India, and determine the scale of regulation and censorship required.

4. In 2016, Mukesh Ambani, India’s richest petrochemical tycoon, launched a telecom network, Reliance Jio, with a $22.5 billion investment, which brought mobile broadband to massive sections of the population, through marketing schemes offering rock-bottom data prices. Also see, Mukherjee (2019).
